# Assessment of Uinta Basin Oil and Natural Gas Well Pad Pneumatic Controller Emissions**

**DOI:** 10.4236/jep.2017.84029

**Published:** 2017-04

**Authors:** Eben D. Thoma, Parikshit Deshmukh, Russell Logan, Michael Stovern, Chris Dresser, Halley L. Brantley

**Affiliations:** 1 Office of Research and Development, National Risk Management Research Laboratory, U.S. EPA, Durham, NC, USA; 2 Jacobs Technology Inc., Durham, NC, USA; 3 Region 8, U.S. EPA, Denver, CO, USA; 4 ORISE Participant, U.S. EPA Office of Research and Development, National Risk Management Research Laboratory, Durham, NC, USA

**Keywords:** Pneumatic Controller Emissions, Oil and Natural Gas Production, Uinta Basin, Methane, Volatile Organic Compounds

## Abstract

In the fall of 2016, a field study was conducted in the Uinta Basin Utah to improve information on oil and natural gas well pad pneumatic controllers (PCs). A total of 80 PC systems at five oil sites (supporting six wells) and three gas sites (supporting 12 wells) were surveyed, and emissions data were produced using a combination of measurements and engineering emission estimates. Ninety-six percent of the PCs surveyed were low actuation frequency intermittent vent type. The overall whole gas emission rate for the study was estimated at 0.36 scf/h with the majority of emissions occurring from three continuous vent PCs (1.0 scf/h average) and eleven (14%) malfunctioning intermittent vent PC systems (1.6 scf/h average). Oil sites employed, on average 10.3 PC systems per well compared to 1.5 for gas sites. Oil and gas sites had group average PC emission rates of 0.28 scf/h and 0.67 scf/h, respectively, with this difference due in part to site selection procedures. The PC system types encountered, the engineering emissions estimate approach, and comparisons to measurements are described. Survey methods included identification of malfunctioning PC systems and emission measurements with augmented high volume sampling and installed mass flow meters, each providing a somewhat different picture of emissions that are elucidated through example cases.

## 1. Introduction

Oil and natural gas (ONG) well pad operations employ natural gas (NG)-driven pneumatic controllers (PCs) for production process control and safety functions [1]. As part of regular operations, typical well pad PCs are designed to emit a small quantity of NG to the atmosphere. Because of the large numbers of PCs in use, methane (CH_4_) emissions associated with this source category contribute significantly to total greenhouse gas (GHG) emissions for the ONG sector. Currently, pneumatic devices, including PCs, account for well over 30 percent of methane emissions, making them among the largest source categories in ONG production field operations [2]. Since the emitted field NG contains a small percentage of higher chain hydrocarbons, PCs also contribute to volatile organic compound (VOC) emissions for the sector. To support environmentally responsible development of these U.S. energy assets, it is of ongoing importance to continually improve information on the number, type, operational conditions, and emissions of well pad PCs, as well as methods to characterize these emissions.

In the fall of 2016, an on-site study of 80 PC systems on eight Utah ONG well pads was conducted in cooperation with three Uinta Basin operators. The goals of the limited-scope effort were to build on existing PC emission research performed in other ONG basins [3–6], help advance PC emissions survey and measurement methods, and inform Uinta emissions inventories to the extent possible. The procedures used in this project were adapted in part from the Oklahoma Independent Petroleum Association (OIPA) study [3], that cataloged 680 PCs on 162 Oklahoma sites using on-site surveys and engineering emission estimates (EEEs), and Allen et al. [5], who sampled 125 PCs in the Rocky Mountain (RM) region, and many more in other ONG basins, using measurement methods similar to methods employed here. Following an introduction to PC types, emission survey and measurement methods are described, and field results are discussed and compared to other studies. In addition to the attached Supplemental Files (SFs), further information on project quality assurance (QA) and data can be found in Archive Files (AF) on the U.S. Environmental Protection Agency (EPA), Office of Research and Development (ORD) Science Hub [7].

Well pad PCs convert a sensed process variable (e.g. mechanical float level, temperature, gas flow, pressure) to a pneumatic valve actuation to control a process or execute a safety function. The expected air emission profile of an NG-driven PC system depends on its design and physical dimensions, the process application and the characteristics of the well pad and product, and on the maintenance state of the PC system. Some NG-driven PCs are designed not to emit to the atmosphere, and a growing number of well pad safety and control value actuation systems are electronically sensed and electrically controlled, producing no NG emissions. These categories of control devices are not considered in this project, nor are other pneumatic devices such as chemical injection pumps, tank pressure relief devices, or non-venting pressure regulators. The potential secondary emissions effects of malfunctioning PC systems are not considered. General fugitive or vented emissions from well pad systems are not part of this study, but when found during field measurements were noted to the operator so corrective action could be pursued if necessary.

In a simplified form, there are four major categories of NG-emitting PCs with definitions based on the combination of the system depressurization method and service type [1]. The depressurization method of a PC relates to its primary venting mode and can be either continuous (CPC) or intermittent (IPC) in time. As part of normal operation, a CPC emits at a relatively constant rate that is modulated or temporarily spiked during the actuation event, (e.g. to open or close a valve in response to a process signal). An IPC has a physical barrier between the NG supply gas and the atmosphere and emits primarily in short bursts, typically a few seconds in duration as part of each actuation event.

**Figure 1.**
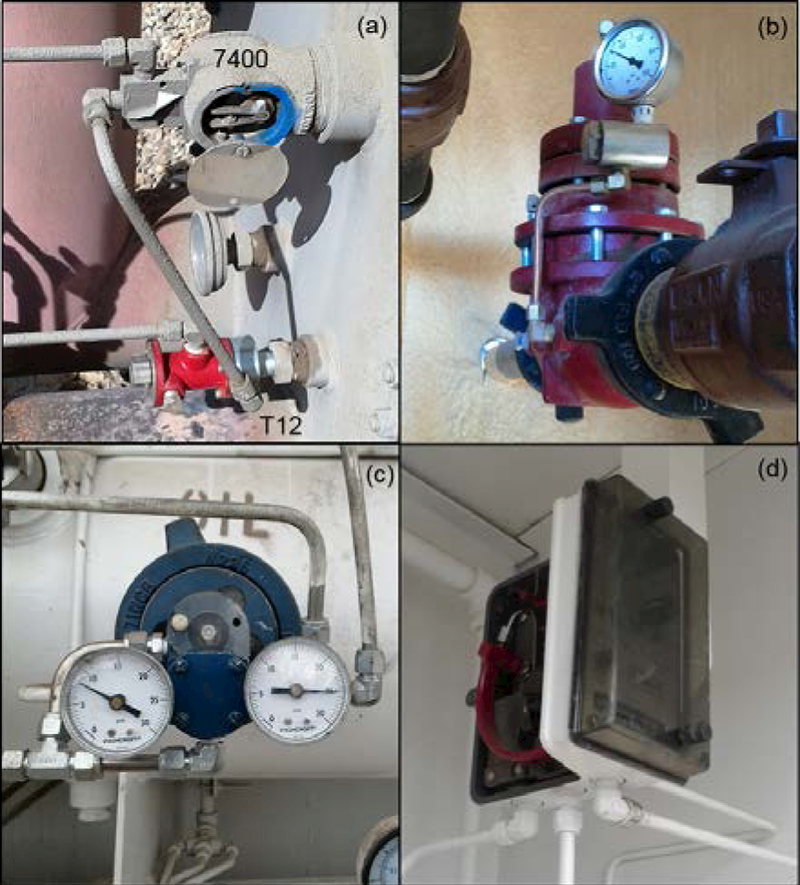
Example PCs encountered in this study: (a) WellMark 7400 IPC level and Kimray T12 temperature IPC, (b) Kimray back pressure regulator IPC, (C) WellMark 6900 level controller IPC, and (d) Fisher™ 4660 high-low pressure pilot CPC with side cover removed.

For each depressurization method, there are two primary PC service types that relate to the degree of valve actuation, and hence the amount of NG released. Some well pad processes require valves to be actuated in a fully “on/off” fashion, whereas other processes require a “throttling” action where the valve set point varies in response to the control loop signal. The amount of NG released during the actuation event of an on/off controller is approximated by the entire volume space of the PC system (including the PC, the valve actuator bonnet, and the connecting tubing), whereas a throttling controller represents a fraction of this volume. Within these four major categories, there are several possible PC subtypes, hybrid combinations, add-on relays, and retrofit packages (designed to reduce emissions from high-bleed CPCs), but these variations were not a factor in the current study so are not discussed. Representative examples of PCs encountered in this study are shown in Figure 1.

From an emissions assessment standpoint, CPC and IPC systems can be viewed as possessing both a constant and a periodic emissions component, with the latter associated with the actuation event. For CPCs, the relatively constant component of emissions is determined by the engineered orifice size and is called the “bleed rate”. The constant component of emissions for an IPC is called the “seepage rate” and is present because it is not possible to make metal to metal seals completely tight under real world conditions [1]. The seepage rate for properly maintained IPC systems should be very low, on the order of 0.05 standard cubic feet per hour (scf/h), as specified by one manufacturer [3]. For both CPCs and IPCs, it also necessary to know the emissions associated with the periodic, short duration actuation events as well as the frequency of occurrence of these events.

Superimposed on the designed emissions are emissions associated with the maintenance state of the PC system. If a PC is not well-maintained or malfunctioning, the emissions from the system can increase significantly. For example, the designed seepage rate of an IPC system may be very low, but the pilot seal quality may degrade overtime due to routine use or debris, causing continuous emissions through the PC exhaust port (or weep hole) that are orders of magnitude higher than the designed rate. Malfunctions can also manifest as emissions from failures in seals or diaphragms in other parts of the PC body or actuator or could be caused by issues with the process the PC is designed to control.

For any analysis of PC emissions, it is critical to define the components that make up the system. The part of the PC system that controls action is called here the “pilot”, and the subsystem that executes action is called the “actuator” and includes the valve effecting the process change. If the pilot and actuator are contained in the same housing, the PC system is called “integral”. If the pilot and actuator are physically separated it is referred to here as a “pilot-actuator PC”. For this analysis, emissions from any subsystem, including the tubing connecting the pilot and actuator, are considered PC system emissions. This inclusive definition is necessary to elucidate differences in measurement methods and is informative for other reasons, but may not comport with regulatory definitions that could ascribe some of these emissions to general fugitives or equipment leaks.

Regarding emission factors (EFs) for PC systems, a large range exists with the U.S. EPA’s Greenhouse Gas Inventory using whole gas device emission rates of 37.3 scf/h and 1.39 scf/h for high and low bleed CPCs, respectively, and 13.5 scf/h for IPCs [8]. The U.S. EPA also defines the maximum emission rate for well pad CPCs as 6 scf/h under 40 CFR Part 60 Subpart OOOO Standards [9]. Considering studies in basins near Utah, OIPA [3] calculated average emission rates of 21.54 scf/h and 0.40 scf/h for CPCs and IPCs, respectively, with an overall average of 1.05 scf/h for Oklahoma sites. With flow meter measurements in the RM region, Allen et al. [5] found 7.23 scf/h and 0.31 scf/h for CPCs and IPCs respectively with an overall average of 0.8 scf/h. These RM region values were significantly lower than Allen’s measurements in other U.S regions with differing production profiles, and also lower than the Prasino Group’s study in British Columbia [6]. Driven by basin-specific product extraction and processing demands, well pad engineering differences clearly play a major role in determining regional PC EFs.

The Uinta basin has both NG and waxy crude oil production well pads with process engineering and PC populations potentially dissimilar to each other and to other basins. In this study, a multistep on-site survey approach was used to gather information on PC populations, assess maintenance states, and execute emissions measurements. In addition to gaining insight on Uinta Basin PC emission profiles, the effort provided some perspective on assessment of PC systems with less invasive tools readily available to operators and inspectors. Use of installed flow meter measurements in both supply line and exhaust port configurations provided additional information on PC system emissions and data for comparisons to EEEs and to other studies. The variety of measurement approaches utilized allowed the strengths and weaknesses of the methods and definitional aspects of PC systems emissions to be explored.

## 2. Methods

### 2.1. Site Description

The cooperating Uinta Basin ONG operators selected the sites that were surveyed and provided site access and on-site technical support for the project. A total of five waxy crude oil well pads, supporting six oil wells in total, and three NG gas well pads, supporting 12 gas wells in total, were surveyed over six field days. Each company used their own site selection criteria with most sites considered to be relatively well-maintained and subject to regular company inspections, as defined by each company’s policy, with inspection frequencies ranging from weeks to months. One of the eight well pads (Gas Site 3) was intentionally chosen to be an older site, without benefit of recent company inspection. The selection process did not systematically consider the regulatory or permit status of the sites. Table 1 provides information on the well pads that were surveyed including the number of wells, date of first production for the first and last wells on site and the cumulative production of oil, produced water, and NG in thousands of barrels (Mbbls) and standard cubic feet (Mscf).

Each of the oil well pad sites sent their produced field gas, (referred to as “sales gas”), for off-site drying, then returned the majority of this processed gas (now called “fuel gas”) to the well pads to operate the PCs and other process functions, such as heaters. Some PCs associated with the separators or (heater treaters) on the oil sites emitted sales gas directly. The gas well pad sites utilized sales gas tapped off the driest part of the process (e.g., highest point of liquids separator) to directly operate PCs and other functions.

### 2.2. PC System Assessment Methods

This study employed QA-augmented versions of methods used in previous studies in other ONG basins [3–5]. The on-site PC assessment procedures consisted of: (1) information gathering, (2) emissions screening with optical gas imaging (OGI) and hand-held probe (HHP) emissions detection, (3) emissions measurement with augmented high volume sampling (HVS) and installed mass flow meters (MFMs), and (4) calculation of EEEs for the PC systems that were encountered. All instrumentation was calibrated to and/or checked with certified high purity CH_4_ (various manufacturers). Whole gas emission rates were measured for a subset of the PC systems with instrument response corrected for gas composition differences and other factors and reported for standard conditions of 20.0 °C and 1013.25 hPa (SF1). Additional information on auxiliary equipment and procedures can be found in AF [7]. The equipment used in this study was not uniformly certified as intrinsically safe for use in hazardous environments. Extreme caution, pre-operation emission safety checks, personal safety monitors, and operator oversight were used to ensure safe operations.

**Figure 2.**
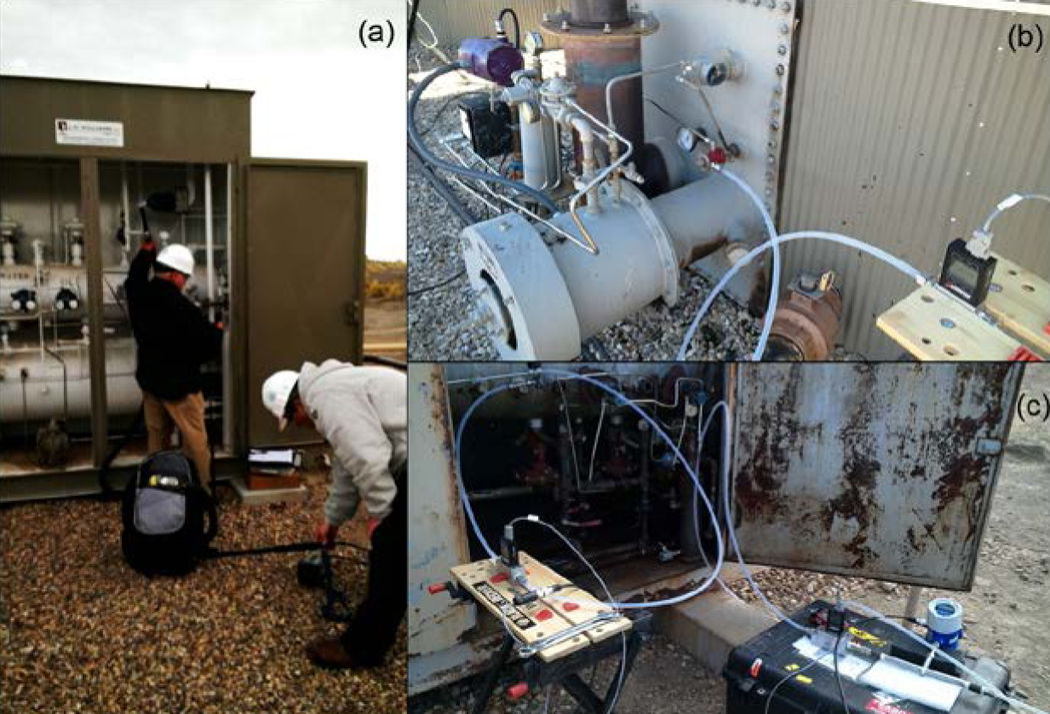
Examples of HVS and installed MFM measurements (a) HVS of Fisher™ 4660 CPC with HHP exhaust check, (b) Alicat MFM installed on exhaust port of Kimray T12 IPC, (c) supply line measurement of two WellMark 6900 IPCs with Alicat and Fox MFMs in series (right), and a Fisher™ 4660 CPC with an Alicat MFM (left).

(1) Information Gathering: Details of PC systems and their functions were gathered through on-site survey and discussion with the ONG operators. Photos of the PC systems and process information such as supply pressures (where possible) were acquired. The PC systems were identified as pilot-actuator or integral type and the manufacturer, model, depressurization method (CPC or IPC), and service type (on/off or throttle) of the PC system was determined. The physical dimensions of the PC system components and connecting tubing were measured for calculation of EEEs (SF1, Data1).
(2) Emissions Screening: Both standard and high sensitivity mode OGI observation of each PC system was conducted using a FLIR GF320 (FLIR Inc., North Billerica, MA, USA). General information on OGI id discussed elsewhere [10]. HHP leak detection of each PC system was performed, primarily using a PPM Gas Surveyor 500 (Gas Measurements Instruments, Ltd., Renfrew, Scotland), with a TVA-1000B (Thermo Scientific, Waltham, MA, USA), and other HHPs [7] were additionally used in some cases. With close in (short distance) inspection, under low effective wind speeds (inside equipment cabinets and sheds), OGI is believed to have routinely detected PC emissions at 1.0 scf/h and above for this project. On-component inspection with parts per million (ppm) sensitivity HHPs routinely detected emissions < 0.1 scf/h. These direct HHP observations did not represent an emissions measurement and cannot be compared to leak detection limits used in other regulatory and compliance programs as direct coupling of an HHP to the exhaust port of the PC can yield an extremely sensitive emission detection, potentially of designed seepage rates.
(3) HVS Emissions Measurements: For PC systems that exhibited continuous emissions in Step (2), an augmented HVS emissions measurement was performed using a Bacharach Hi Flow^®^ Sampler (Bacharach, Inc., New Kensington, PA, USA). This instrument was the only commercially available HVS for NG measurements and is described elsewhere [4,5, 11–13]. The augmented QA protocol was necessitated by previous observations of potential HVS malfunction [11–13] when measuring mixed hydrocarbon (HC) emission streams. The protocol included a pre-deployment instrument update and check-out by the manufacturer, standard HVS sensor calibrations (typically daily with 2.5% and 100% CH_4_), multiple in-field mass flow controller-based simulated emission tests to confirm overall HVS operation, and 100% HVS exhaust stream checks with the HHP to confirm each measurement. Figure 2(a) illustrates an HVS measurement of a CPC with the HHP exhaust stream QA check. With a maximum of 1.9% and an average of 0.3% HVS exhaust stream HC concentration, all field HVS measurements were well below the previously observed sensor transition failure level of approximately 5% HC concentration, thus eliminating a major source of HVS uncertainty for this particular data set. Simultaneous OGI was used to ensure plume capture in most cases. The minimum quantification limit (MQL) of the augmented HVS approach was determined to be ≈ 0.2 scf/h, using the HHP probe for readings < 0.4 scf/h in some cases (SF1, Figure S1). The uncertainty of the HVS approach was estimated at ± 30% with correction factors for mixed HC streams and instrument flow rates ranging from +10% to +22% (SF1, Data2). Due to relatively low sampling rates (~ 0.3 Hz) and multi-second stabilization times, the HVS was limited to measurement of relatively continuous emissions and was not used to assess the rapid (few second) manual PC actuation trials in this study. For instrument response correction purposes, representative evacuated canister grab samples (1.4L Silonite® 29-MC1400SQT, Entech Instruments, Simi Valley, CA, USA), were acquired at the exhaust port of the HVS and analyzed for speciated non-CH_4_ organic compounds using EPA Method TO-1A [14] and CH_4_ by EPA Method 18 [15], (SF1, Data2; AF [7]).
(4) MFM Emissions Measurements: Following Steps (1), (2), and (3), emissions from a subset of PC systems were measured using one or more installed MFMs [MW500SLPM, MW100SLPM, MW10SLPM (Alicat Scientific, Inc., Tucson, AZ, USA), FT3 (Fox Thermal Instruments, Marina CA, USA)], subsequently referred to as A500, A100, A10, and FT3, respectively. Data were recorded using a custom data acquisition system operating at 1 Hz (Techstar Inc. Deer Park, TX, USA). The system allowed simultaneous recording of all four MFMs and was fitted with 30 m cables so multiple areas of the well pad could be monitored. The Alicat MFMs were fast response (<100 ms), low pressure-drop laminar flow meters while the FT3 was a thermal MFM with 1–3 seconds response times with a similar model used by Allen et al. [5]. The FT3 was factory calibrated to CH_4_ in two operational ranges, zero to 100 scf/h and zero to 500 scf/h, but only the former range was used. The MQLs of the MFMs were determined to be ≈ 0.5 scf/h, ≈ 0.2 scf/hr, <0.1 scf/h, and ≈ 2.0 scf/h for the A500, A100, A10, and FT3, respectively, with an uncertainty estimate of ± 10% with low bias increasing as MQL was approached for the A500 and FT3 (SF1, Figure S1). Instrument correction factors for mixed HC streams ranged from +2% to +7% for the A500, A100, and A10 and +3% to +10% for the FT3 (SF1, Data2). The MFMs and HVS were checked with calibrated flow controllers before, after, and multiple times during the study with simulated emissions ranging from 0.1 scf/h to 160 scf/h and primary check points of 5.0 scf/h and 50.0 scf/h.
The MFMs were installed on either the exhaust port of the PC pilot [Figure 2(b)] or on the supply lines of one or more PCs systems [Figure 2(c)]. Exhaust port monitoring, which was not possible in all cases due to PC design, provided a focused view of the PC pilot emissions and was the only practical choice for MFM measurements of integral PC systems [e.g., Figure 1(b)]. Supply line measurements provided a more comprehensive view of the PC system, including emissions from connecting tubing and actuators, and potentially unrelated well pad systems present on the common supply. Following previous method critiques [9, 11] the HVS measurement was used prior to installation of the MFMs to avoid inadvertent PC malfunction reset during installation. The installed MFM tubing connections were leak-checked with OGI, HHD, and/or a soap liquid bubble test, and care was taken by the operator to set the supply pressure to ‘as found’ conditions. The MFM emissions measurements were typically performed for a time period of more than one hour in an attempt to observe the natural actuation rate of intermittent PCs. In many cases, manual actuation experiments were conducted at the end of the measurement cycle to produce data for comparison to EEEs. Unless otherwise specified, all MFM values reported here are from the Alicat models.
(5) Engineering Emission Estimates: Similar to the procedures used by OIPA [3], EEEs were developed for the PC systems that were encountered (SF1 Data1). A simplified emissions estimate equation (E) for each PC system was utilized:

Eq. 1 E =Ce+Ae*Ar

where: *Ce* = Continuous emission rate from PC system in scf/h

*Ae* = Emissions associated with a PC system actuation event in scf

*Ar* = Number of PC system actuation events occurring in one hour

As previously discussed, for CPCs, *Ce* is the orifice size-determined bleed rate with actuations appearing as a discrete or near-continuous modulation of this rate. For IPCs, *Ce* is the designed seepage rate for well-maintained systems and is conservatively assumed here to be 0.1 scf/h. This level is 50% of the HVS MQL and is easily detected by HHP. Continuous emissions from malfunctions of the PC system are also part of *Ce,* appearing primarily as an additive term to the designed *Ce,* but may also affect *Ae*. Using the physical dimensions of the measured connecting tubing, actuator model measurements and information, and process data, the physical volume of the actuators and the emissions per actuation event for the PC system were determined using a simplified ideal gas law approach developed by Kimray (AF [7]). Whereas OIPA [3] assumed four actuations per hour for infrequently actuating IPCs, this analysis employs five potential discrete actuation frequency bins for the PC systems; 1/month, 1/day, 1/h, 4/h, or 20/h. Consistent and rapidly actuating CPCs are assumed to appear as a modulated continuous emission with average emission rate values determined by measurement and safely ascribed to the *Ce.* In each case, a more frequent actuation bin is used for the EEE. For example, a tank temperature controller may actuate once per day in the summer or several times der day in the winter but a once per hour (1/h) bin is assumed in the EEE. Some information on natural PC actuation rates was available from aural observations (e.g. audible flow, burners firing, etc.), the MFM measurements of Step (4), and through limited trial application of simple diaphragm mechanical counters (SERN-5, Control Equipment Inc. Wichita Falls, TX, USA).

## 3. Results and Discussion

### 3.1. PC Types Encountered

A total of 80 in-service NG-emitting PC systems were found on the eight well pad sites and those PC systems were subjected to the survey procedures described in Section 2. Table 2 summarizes the total number of PCs, the number classified as IPCs, the average number of PCs per well, and the three most frequently encountered PCs on each site. Only three PC systems (4%) were classified as CPCs. Eighty-eight percent (88%) of the PC systems were identified as snap-action on/off controllers with the remainder the throttling variety. Fifty-eight percent (58%) of the PCs were used for process control with the reminder serving a safety or process protection function. Fifty percent (50%) of the PCs were associated with separators or heater treaters, 38% with tanks, 6% with enclosed combustors, and 6% with well control. Forty-five percent (45%) of the PC systems controlled or monitored liquid levels, 35% temperature, and 20% pressure.

The three continuous PCs were Fisher™ model 4660 high-low pressure pilots (Emerson Electric Co., St. Louis, MO, USA) that actuated safety shut-in valves on each of the gas sites [Figure 1(d)]. Kimray model T12 temperature controllers (Kimray Inc., Oklahoma City, OK, USA) accounted for 35% of PCs encountered and were used for separator and tank burner control and temperature-related process protection functions [Figure 1(a)]. The WellMark 7400 Snaptrol level controller (WellMark LLC, Oklahoma City OK, USA) also accounted for 35% of the PCs surveyed and were frequently found in series with the Kimray T12 PCs, usually providing a tank or vessel liquid level burner shutoff protection in these cases [Figure 1(a)].

The WellMark 6900 snap-action level controller was prevalent at the gas sites accounting for 41% of the PCs on those well pads and 9% of the overall total. The four aforementioned PC types were of the pilot-actuator variety, which accounted for 89% of the PC systems surveyed. The last commonly encountered PC was the Kimray back pressure (BP) controller (10% of occurrences), which is an integral PC. Five of the remaining six PC systems were of the pilot-actuator type including three electronically controlled oil well shut-in safety devices, a “direct to sales” gas metering system, and an unidentified level controller. The last PC was an integral type Kimray venting pressure regulator.

The high percentage of IPC systems observed was similar to the percentage reported in the OIPA study [3] that found 97% of the 680 PCs surveyed were of the intermittent vent variety. In the RM region, Allen et al. [5] sampled 125 PCs, primarily from NG and condensate- producing sites (likely not including waxy crude oil sites), finding 91% IPCs. Allen et al. employed a measurement-based type assignment for continuous PCs that could include constantly emitting IPCs that are classified as malfunctions in this analysis. The most notable difference in the OIPA PC populations was that BP controllers were the dominant PC type (40%), a much higher percentage than we observed in the current study. This difference is believed to be primarily due to site production and engineering design factors, such as generally less need for gas pressure management, and the requirement to maintain a minimum crude temperature for waxy crude at the Uinta Basin oil sites. Assuming the population of PCs measured in this study is representative of the basin, the difference between oil and gas site engineering is reflected in the large differences in the average number of PCs per well, 10.3 for oil sites, compared to 1.5 for gas sites. The latter may be depressed for the specific sites surveyed in comparison to the basin-average for gas sites as no plunger lift controllers were found and the front end well pressure control was accomplished with electrically actuated devices. The OIPA study found an even lower number of 0.85 PCs per well on average while Allen et al. found 2.7 PCs per well for all regions, making the high number of PCs per well at the Uinta oil sites noteworthy.

### 3.2. Continuous Emissions and Malfunctions

For this analysis, a PC system is defined as all control loop components necessary to execute the process or safety function. Any significant (non-designed) emission, such as a leaking PC body, tubing connector, or actuator diaphragm, is considered a PC system maintenance issue or a malfunction (terms used interchangeably). Screening detects were expected and observed on the three CPCs (Fisher™ 4460). Since the 77 IPCs had low expected actuation periods (>15 minutes), sustained emissions observed with OGI and HHP and verified with HVS and/or MFM to exceed 0.2 scf/h, were defined as malfunctioning PC systems. Table 3 summarizes the emissions assessment surveys with focus on those identified as malfunctioning. Here the three continuous Fisher™ 4460 PCs at Gas Sites 1–3 are included in the HHP and OGI detection counts, but the HVS-measured emission rates (0.8 scf/h, 2.2 scf/h, and 0.3 scf/h, respectively) are assumed to be designed CPC venting and are therefore not included in malfunctioning PC emission rate column.

**Figure 3.**
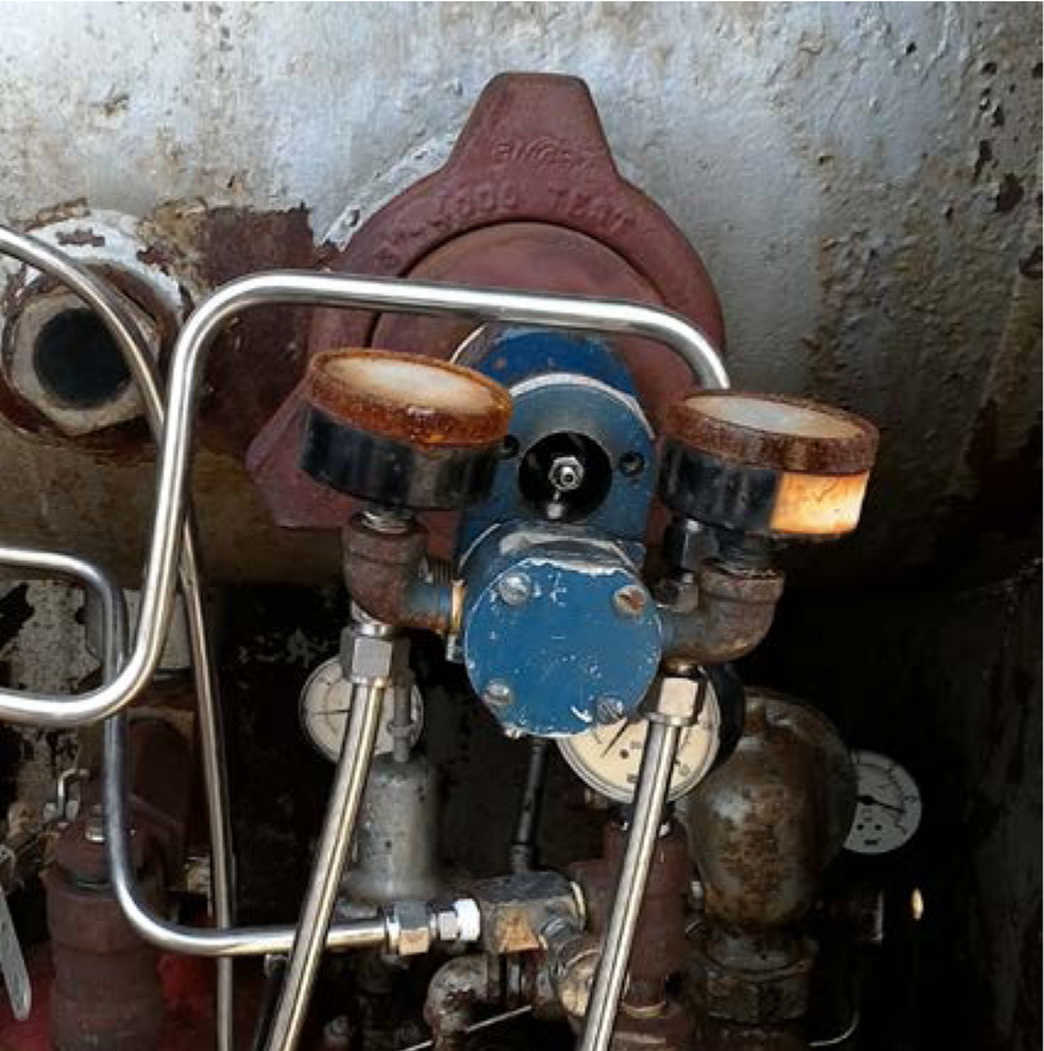
An older WellMark 6900 IPC found to be malfunctioning.

As expected, the HHP screen yielded a higher number of potential malfunction detects (28%) compared to OGI (16%), as even small emissions can be found with ppm-sensitivity HHP probes. Out of the 20 HHP detects on IPCs, 11 exhibited continuous emissions above the defined 0.2 scf/h malfunction level, as measured by HVS, with all of these detected by OGI. Malfunctioning IPCs exhibited emission rates ranging from 0.3 scf/h to 4.5 scf/h, with an average of 1.6 scf/h. The rate of PC malfunctions was 14.3 % for this study, 8.2 % for oil sites and 28% for gas sites; however, this difference is in part due to site selection factors. The malfunction rate varied widely over the sites from 0% to 60%. Three of the eight sites appeared to be very well maintained with a higher percentage of newer looking PCs and no observed malfunctioning PCs systems. Gas Site 3, an intentionally selected older, less frequently inspected site, had the highest percentage of malfunctioning PCs and possessed an older WellMark 6900 water dump valve controller (Figure 3) that exhibited the highest observed study emissions at 4.5 scf/h, and was found to be non-operational (would not actuate a dump) in manual actuation trials.

An important factor in the on-site survey assessment of the maintenance states of PCs and identification of the origin of emissions is related to the ability to observe and access the entire PC system. The ‘entire PC System’ refers to all normally external components, such as the pilot, actuator, and connecting tubing. In theory, part of the PC system, for temperature or level controls for example, extends inside the tank or vessel and may provide a path for emissions, but this route is not considered here. Starting with easily understood cases, some of the complexities of the PC system assessment and the advantages and disadvantages of particular measurement approaches are discussed.

Two straightforward examples of Table 3 were the WellMark 7400 (actuator) cases (Oil Sites 2 and 5), which had emissions originating from the valve stem travel indicator of the Kimray 212 SMA PO actuator. In these cases, the entire PC system was fully observable and the emissions were easily identified by OGI and HHD, and measured by HVS. These malfunctions were repaired by the operator in a very rapid fashion by tightening the travel indicator, and the actuator was then confirmed to be emission free. Under other definitions, these emissions could be considered equipment leaks and not part of the PC system.

For the oil sites, Kimray T12 and WellMark 7400 PC systems [Figure 1(a)] commonly supported storage tank heating functions, accounting for 38% of all PCs and were easily observable and accessible. On Oil Site 3, two Kimray T12 tank burner controllers were emitting from the exhaust port and potentially the body of the controllers with HVS measurements of 1.4 scf/h and 3.4 scf/h for these units. Exhaust port MFM measurements of the same units were 1.0 scf/h and 1.7 scf/h, respectively, indicating that only a portion of the emissions were exiting the exhaust port and could be assessed with this MFM approach.

On Oil Site 5, the Kimray T12 horizontal separator temperature controller and an in-series WellMark 7400 fluid level safety shut-off PC were part of a complex system that once utilized a secondary burner control package (now by-passed) and had extended lengths of tubing, much of which was hidden inside the separator vessel insulation. MFM supply line measurements showed that this combined system had a continuous emission rate of 3.4 scf/h but HVS measurements could detect only 0.3 scf/h emissions from the body of the WellMark 7400 and were below detection limit on the Kimray T12. This complex system shared supply lines with several PCs, also with partially hidden lengths of tubing. An unidentified, emission point was present but could not be located so the majority of MFM-determined emissions were arbitrarily assigned to the Kimray T12 (3.1 scf/h, Table 3). An HVS-only or exhaust port MFM measurement would underestimate PC system emissions in this case.

In a similar case, all PC system components of the malfunctioning Gas Site 3 WellMark 6900 dump valve (Figure 3) could not be assessed fully because the actuators were confined in the back of the cabinet. Even though this PC would not actuate (operational malfunction), and produced the highest emission rate as determined by MFM supply line measurements (4.5 scf/h), the initial HVS measurement indicated a 0.2 scf/h emission from the pilot, which changed as the operator attempted adjustments to regain functionality (subsequent to the initial MFM measurement). The operator could affect (and reduce to low levels) the supply line flow registered by the MFM pair [Figure 2(c), right] by adjusting the pilot all the way out but was unsuccessful in restoring functionality, and a later rebuild was required. The original point of emissions was uncertain, and it is possible the origin point was internal to the vessel.

In addition to PC component access, temporal variability in the PC system malfunction states must also be considered. As an example, at site Gas 2, a WellMark 6900 was found initially to be malfunctioning at a rate of 1.6 scf/h, but after several trial actuations, this rate was reduced without other adjustment to below 0.2 scf/h. Variable emissions were subsequently measured (0.3 to 1.3 scf/h) and we observed that even a small motion of the connecting tubing for this controller could change the continuous emission level of this PC. In another case, an experiment was performed on an infrequently actuated over-pressure protection PC. This PC was not emitting prior to the experiment but after a trial actuation, the PC pilot did not seal properly, likely due to long accumulated debris. An additional actuation cleared the issue and emission ceased. Similar behavior was observed in other cases, and it was evident that for these sites, manual actuation of some types of PCs was routinely performed by the operators to induce resets of potentially malfunctioning states or to check PC functionally.

Regarding the continuous emission (*Ce*) term of Eq. 1, out of the 80 PC systems, 14 exhibited measureable continuous emissions ranging from 0.3 scf/h to 4.5 scf/ h, with an average of 1.5 scf/h (includes three CPCs). The remaining 66 IPC systems passed the HHD, OGI and emissions measurement screen and were assigned a *Ce* of 0.1 scf/h for this analysis. Allen et al. [5], produced 15-minute duration HVS and MFM measurements of all 125 PCs in the RM region with 22% of these PCs exhibiting whole gas emission rates > 0.1 scf/h as compared to 18% for the current study. The average whole gas emission rate measured by Allen was 0.8 scf/h with 13% of readings exceeding 1.0 scf/h with highest three readings of 11.3, 13.8, and 19.8 scf/h. The largest PC emissions levels observed by Allen et al. were significantly higher than the levels observed in this study and this difference may be due to a combination of the site types (oil vs. gas), engineering utilized (e.g., lack of high-bleed continuous controllers), gas production levels, and operator inspection and maintenance practices for sites surveyed.

### 3.3. Actuation Event Emissions

In addition to *Ce* of Eq. 1, the emissions per actuation event *Ae* and event occurrence rate *Ar* must be understood. For the latter, from operator input and aural observations, it was apparent that the expected actuation frequency of a large portion of the encountered PC population was very low. In an attempt to observe naturally occurring actuation rates, the installed MFM measurements were conducted for an extended observation period of one hour, longer than the 15-minute observation period used in previous studies [3, 5], with no natural actuation events registered. Five representative Kimray T12 PC systems were monitored with SERN-5 pneumatic counters for approximately one month with data further supporting low (multi-hour) actuation rates. For the current data, only three of the five actuation frequency bins for IPCs described in Section 3.2 (Step 5) were required. Approximately 50% of the PC systems were conservatively assumed to actuate once per hour. Evidence suggested 16% of the PC systems actuated daily to several times per month (e.g. secondary separator or liquid knockouts, low tank level PCs), assumed here to be once per day. The remainder of the PC systems were safety or process protection devices with extremely infrequent actuations, assumed here to be once per month.

A total of 18 IPCs and one CPC were measured with installed MFMs, and manual actuation experiments were conducted successfully in 14 cases. Six Kimray T12 tank burner controllers [Figure 2(b)], two Kimray T12 separator or bath burner controllers, and 3 Kimray BP controllers were measured using exhaust port MFM sampling. MFMs were installed on the supply lines of four Wellmark 6900 dump valves (two sets of two in series) [Figure 2(c)], a T12 separator burner controller in series with a Wellmark 7400, a WellMark level controller, and a Fisher™ safety shut-in controller [Figure 2(c)]. In some cases, multiple MFMs were installed in series for comparison purposes.

**Figure 4.**
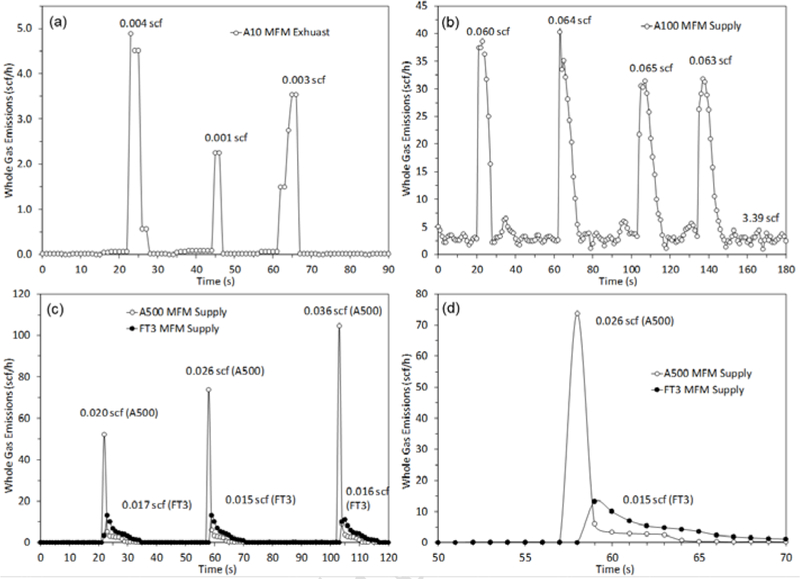
Manual actuation experiment of (a) Kimray T12 tank burner IPC, (b) complex Kimray T12 Separator IPC with malfunction, (c) Wellmark 6900 dump valve with both Alicat and Fox MFMs, and (d) Expanded view of (c).

Figure 4 shows several examples of repeat actuation experiments with emissions for individual actuations determined by the trapezoidal rule integration method with baseline line removed using code written in “R” [16]. These actuation experiments were conducted by the operator triggering a manual dump in the case of a Well Mark 6900 dump valve PC, or turning the temperature set point up and down for a Kimray T12 temperature controller. The very fast (3 s to 5 s) and low emission response (< 0.01 scf) of the T12 in Figure 4(a), is typical of many PC systems encountered in this study. The low emissions of Figure 1(a) are due to the small actuation volume of the Kimray 112 SMT DAB motor valve (~ 1.1 in^3^) and limited tubing lengths, a very common scenario with 60% of all PC systems surveyed using this actuator. Figure 4(b) shows actuation events for the previously discussed Oil Site 5 malfunction case (Kimray T12, WellMark 7400). With tubing lengths approaching 600 inches, higher than normal supply pressures (50 psi), these actuations were among the highest measured in the study and the baseline (between actuations) exhibited significant variability. Figures 4(c) and 4(d) show supply line-measured actuation experiments of a Wellmark 6900 dump with both Alicat and Fox MFMs. Here the temporal response differences of the meters are evident and could play a factor in the observed differences. In general, the 1 Hz sampling rate of the data acquisition system could be increased to take advantage of the high response rate of the Alicat MFMs and to better characterize the temporal profiles. As a thermal meter, the Fox FT3 would benefit less from higher data acquisition rates.

**Figure 5.**
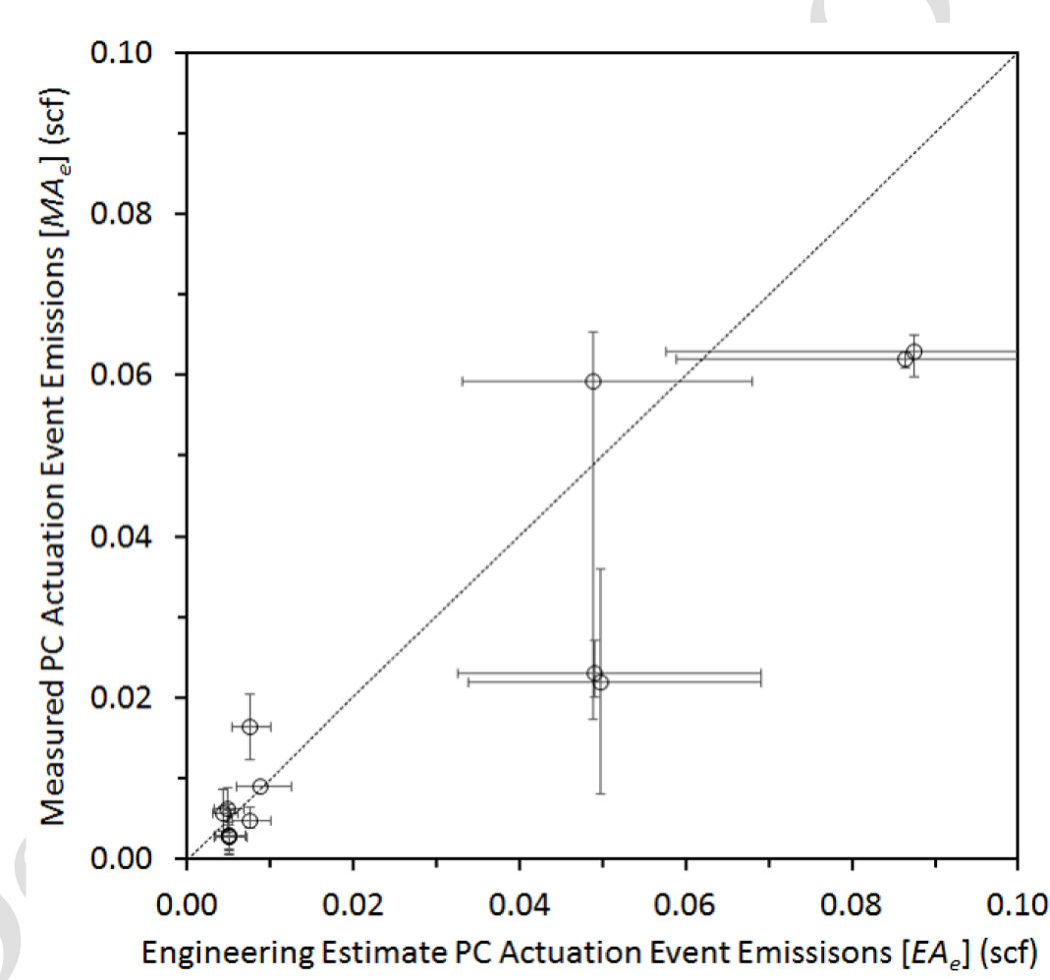
Comparison of EEE (*EAe*) and measured actuation events (*MAe*). Error bars for *EAe* (high/low) represent ± 20% actuation volume, ± 20% pressure, and −/+ 20% temperature based on conditions at the time of the survey. Error bars for *MAe* are minimum and maximum measured values.

Using field conditions at the time of measurement, EEE values for emissions per actuation event (*Ae*) were calculated, and compared to measured actuation events for the 14 available manual actuation trials (Figure 5). Rough magnitude agreement of the calculated (*EAe*) and measured (*MAe*) data is evident. The somewhat higher *EAe* values were likely due in part to the simplified model employed that does not account for gas compressibility or potential entrained liquids. The slow time response of the current MFM data acquisition system [Figure 4(d)] may contribute to the *MAe* low bias. Some of larger outlier values were associated with malfunctioning PCs or installations on complex shared supply lines. The abscissa error bars of Figure 5 illustrate the sensitivity of *EAe* to the 20% level (combined) input parameter changes, with the relatively well-bounded nature of the low actuation volume evident in these PC systems.

### 3.4. Composite Study Results and Comparisons to Other Basins

In a manner similar to OIPA [3], EEEs were produced for each of the PC systems using Equation 1 with *Ce* equal to the measured value in 14 cases (11 malfunctioning IPCs and three CPCs) and set to 0.1 scf/h in the remainder of cases. For *Ae*, the calculated EEEs with annual average conditions were used. For *Ar*, conservative bin estimates for actuation frequencies were used with the following assignments [0% (20/h), 0% (4/h), 49% (1/h), 16% (1/day), and 35% (1/month)]. In two cases (Kimray 312 FGT BP vessel pressure relief safety PCs on Oil Sites 1 and 2), a 12 hr process upset vent of 27.7 scf/h, once per month was assumed, with the level based on an actuation trial that simulated a process upset condition (SF1 Data1).

Using the combined measurement and EEE analysis approach, the study mean whole gas emission rate was estimated at 0.36 scf/h per PC system with a median of 0.11 scf/h and a standard deviation of 0.77 scf/h. Seven values (9%) exceeded 1.0 scf/h with a maximum of 4.58 scf/h with six of these values ascribed to malfunctioning PC systems. The mean and median values reflect the assignment of 0.1 scf/h for *Ce* for IPCs with no detectable emissions, as this assumption defines the low end of the distribution. Due to the small actuation volumes and low actuation rates for a large portion of the population, the Ae*Ar component of Equation 1 plays a small role in the estimate of emissions for the study. As a percentage of emissions for each device, the actuation event portion of the PC system emissions accounts for less than 15% of the emissions in 94% of the cases. To investigate the sensitivity to default *Ar* bins, the mean emission rate was calculated assuming higher actuation frequencies. Use of the conservative values in OIPA[3], four actuations per hour, increased the study mean emission rate to from 0.36 scf/h to 0.54 scf/h. Setting the *Ar* term to 10 actuations per hour produced a study mean of only 0.81 scf/h, further illustrating the potential relative impact of malfunctioning PCs with *Ce* rates exceeding 1.0 scf/h.

With the distribution dominated by IPCs, the current study mean (0.36 scf/h), was comparable to the IPC values from the OIPA study (0.40 scf/h) [3] and the RM region of the Allen et al. (0.31 scf/h) [5]. Including both IPCs and CPCs, the overall average for Allen et al.’s RM region was 0.8 scf/h with the continuous group (9% of PC systems) exhibiting an average of 7.23 scf/h, seven times higher than the CPC average of the current study (1 scf/h). Allen et al. measured wellhead, plunger lift, and dehydration PCs that were not found in this study, potentially explaining some of the difference. In other regions of the U.S., Allen et al. found much higher PC emissions compared to the RM region with an overall study average of 5.5 scf/h with group average emissions of 21.8 scf/h and 2.2 scf/h for CPCs and IPCs, respectively. The Prasino study [6], with focus on higher bleed CPCs, also differs greatly from the current result. The complete lack of high emitting CPCs or malfunctioning IPCs > 6 scf/h in the current study is an obvious difference but trends similarly with the RM region of Allen et al., compared to other study regions (relatively higher liquids production levels in the RM are assumed, as in the Uinta). Overall, Allen noted that 76% of devices with emission rates greater than 6 scf/h were in service on compressors (not part of this study) or as level controllers on separators, with the latter also observed here as a high emitting group.

Breaking down the current study, the overall average whole gas emission rate for the oil sites was 0.28 scf/h compared to 0.67 scf/h for the gas sites, with a much higher relative percentage of PCs emitting greater than 1 scf/h for the latter. From evacuated canister measurements (SF1 Data 1), the approximate average molar percentage [weight percentage] of CH_4_ emissions was 88.2% [75.3%], and 93.7% [85.6%] for oil sites and gas site respectively. Combining the whole gas emission rates and gas profiles, oil site PC systems on average emit 60% less methane than gas site PCs, for this limited scope study. Since malfunctioning PC systems help drive this difference, the observation of lower PC system emissions on oil sites should take into account the relative gas well pad site age (Table 1) and site selection (intentional choice of Gas Site 3, not recently inspected) used in this study. This information coupled with the observation of, on average, 10.3 PCs per well for oil sites, compared to 1.5 PCs per well for gas sites, shows that intra-basin engineering differences on the two types of production sites likely reflect two distinct populations.

## 4. Conclusions

This observational study produced information on 80 PC systems on eight Uinta Basin ONG sites using a combination emission survey and EEE approach. The study was performed in cooperation with three ONG operators who selected the sites. With limited scope and nonrandomized sampling, the degree to which study results are representative of the basin is not known. However, several general conclusions can be drawn. Waxy Crude Oil and NG sites in the Uinta Basin have very different PC population profiles, with the former utilizing a larger number of PCs for heating functions. Overall, the percentage of IPCs in the basin is likely very high and may approach 100% for oil sites. For oil sites in particular, a significant percentage of IPC systems have low actuation volumes and actuation rates with typical designed emissions profiles representing a small fraction of a scf/h.

A significant difference in PC emission levels from oil and NG sites was noted and was likely due in part to company differences in site selection and average age of the sites. A key finding was that the emissions were dominated by malfunctioning PC systems, which were defined here to include actuators and connecting tubing. The emission assessment survey procedures used here centered on identifying IPCs with continuous emissions above 0.2 scf/h, and therefore defined as malfunctioning. Along with CPCs, measurement of continuous emissions from malfunctioning IPCs are critical for understanding the population, with non-malfunctioning IPCs emissions more efficiently determined by EEEs. The HVS and installed MFM exhaust port and supply line measurements each provide value, offering a different picture of emissions with varying degrees of implementation burden. For many fully accessible PC systems encountered in the Uinta Basin (e.g., tank heaters), emissions can be effectively determined using an augmented HVS protocol and EEEs. For PC systems with a higher potential to emit or with inaccessible features, installed MFM measurements likely provide additional emission measurement information.

Due to the high percentage of IPCs and their generally low actuation volumes and rates, the overall emission profile of PC systems in the Uinta Basin was determined in large part by the frequency of occurrence of malfunctioning PC systems. For the definitions employed here, this malfunction rate was found to be 14% with these PC systems emitting at levels four times the study average. Future work in the Uinta Basin should focus on randomized sampling in an attempt to more accurately characterize malfunction rates and levels of emissions.

## Supplementary Material

sup 1

## Figures and Tables

**Table 1.** Site information and cumlative well pad production for the surveyed sites.

Site	No. of Wells	Prod. Start	Oil	Water	Gas
	(N)	(MM/YY)	(Mbbls)	(Mbbls)	(Mscf)
Oil 1	1	11/2012	70.9	100.8	22.4
Oil 2	2	07/2006, 07/2014	96.5	106.1	8.4
Oil 3	1	03/2015	32.3	28.4	7.6
Oil 4	1	04/2015	26.2	33.5	12.0
Oil 5	1	01/2015	88.4	186.2	40.1
Gas 1	4	06/2000, 8/2000	6.3	16.8	2818.9
Gas 2	3	04/1982, 12/1999	9.4	12.0	3300.8
Gas 3	5	08/1983, 12/1998	9.1	17.4	4594.0

**Table 2.** PC type summary by site with intermittent vent (IPCs) accounting for 96% of the total.

Site	PCs	IPCs	PCs per Well	Three Most Common PC Types by Site
	(N)	(N)	(N)	Manufacturer, Model Family, (N)
Oil 1	15	15	15	WellMark 7400 (7), Kimray T12 (4), Kimray BP (3)
Oil 2	14	14	7	Kimray T12 (5), Wellmark 7400 (4), Kimray BP (2)
Oil 3	10	10	10	WellMark 7400 (5), Kimray T12 (4), Kimray BP (1)
Oil 4	12	12	12	WellMark 7400 (6), Kimray T12 (5), Kimray BP (1)
Oil 5	11	11	11	WellMark 7400 (6), Kimray T12 (3), Kimray BP (1)
Gas 1	6	5	1.3	WellMark 6900 (3), Kimray T12 (2), Fisher 4460 (1)
Gas 2	7	6	2.3	Kimray T12 (3), WellMark 6900 (2), Fisher 4460 (1)
Gas 3	5	4	1.0	WellMark 6900 (2), Kimray T12 (2), Fisher 4460 (1)

**Table 3:** Summary PC emission assessment surveys with focus on malfunctions

Site	HHP Detects	OGI Detects	Malf. PCs	Malf. PCs	Malf. PC^1^ Emission rate(s)
	(N, %)	(N, %)	(N, %)	Identity	(scf/h)
Oil 1	0, 0	0, 0	0, 0	N/A	N/A
Oil 2	4, 28	1, 7	1, 7	WellMark 7400 (actuator)	0.7
Oil 3	2, 20	2, 20	2, 20	Kimray T12 (2)	1.4, 3.4
Oil 4	1, 8	0, 0	0, 0	N/A	N/A
Oil 5	6, 55	3, 27	3, 27	Kimray T12, WellMark 7400, WellMark 7400 (actuator)	3.1*^, 0.3, 1.2
Gas 1	1, 17	1, 17	0, 0	N/A	N/A
Gas 2	4, 57	4, 57	2, 29	Kimray T12, WellMark 6900	0.4, 1.6*
Gas 3	5, 100	3, 60	3, 60	Kimray T12, WellMark 6900 (2)	0.3, 4.5*, 0.6

1 Defined as malfunctioning (malf.) if continuous emissions > 0.2 scf/h for IPCs or > 6 scf/hr for CPCs [assumes a low bleed category for continuous PCs (9)]. All measurements were HVS, except

(*) by Alicat MFMs. Emission rates are whole gas at standard conditions with gas stream composition correction factors applied.

(^) Multiple PC systems with hidden tubing, location of emission not identified, 3.1 scf/h arbitrarily assigned to Kimray T12.
